# Untargeted Multiple Reaction Monitoring

**DOI:** 10.1021/acs.analchem.5c06838

**Published:** 2026-03-17

**Authors:** Winnie Uritboonthai, Aries Aisporna, Linh Hoang, Bill Webb, Elizabeth M. Billings, Corey Hoang, Mirna Tobea, Chelsea C. Cates-Gatto, Amanda J. Roberts, Tony Teav, Rebecca Borreggine, Ioana Kyritsi, Hector Gallart-Ayala, Julijana Ivanisevic, Anna Popova, James R. Williamson, Robert Plumb, Gary Siuzdak

**Affiliations:** † Scripps Center for Metabolomics and Mass Spectrometry, The Scripps Research Institute, La Jolla, California 92037, United States; ‡ Gnotobiotic and Cryobank Facility, The Scripps Research Institute, La Jolla, California 92037, United States; § Animal Models Core Facility, The Scripps Research Institute, La Jolla, California 92037, United States; ∥ Metabolomics and Lipidomics Platform, Faculty of Biology and Medicine, University of Lausanne, Lausanne CH-1015, Switzerland; ⊥ Department of Integrative Structural and Computational Biology, Department of Chemistry, The Scripps Research Institute, La Jolla, California 92037, United States; # The Skaggs Institute for Chemical Biology, The Scripps Research Institute, La Jolla, California 92037, United States; ∇ Centre for Metabolomics Research, University of Liverpool, Liverpool L69 7ZB, U.K.

## Abstract

Multiple reaction monitoring (MRM) enables robust and sensitive quantification but traditionally requires predefined precursor–fragment transitions, limiting its use in discovery-driven studies. Here, we describe untargeted/micro/universal multiple reaction monitoring (uMRM), a workflow that converts high-resolution untargeted liquid chromatography–mass spectrometry/MS (LC–MS/MS) data into scheduled triple-quadrupole MRM transitions. Pooled-sample LC–MS and stepped-energy DDA MS/MS acquisitions (0, 10, 20, and 40 eV) are used to capture precursor and fragment information representative of each experimental set. Detected features undergo automated deisotoping and empirically validated in-source fragment filtering, followed by spline-based modeling of collision-energy–dependent fragmentation to define optimized precursor–fragment transitions. Transitions are scheduled using retention times observed in pooled samples and deployed on triple-quadrupole instruments without requiring nonlinear retention-time alignment or authentic standards. Across representative biological matrices, including urine, brain tissue, and cultured cells, uMRM enabled automated generation of quantitative MRM methods from untargeted discovery data. Benchmarking across seven triple-quadrupole platforms demonstrated strong agreement between uMRM-derived and experimentally optimized collision energies. By converting discovery-scale data sets into compact transition tables suitable for quantitative deployment, uMRM provides a reproducible approach for linking untargeted LC–MS/MS acquisition with targeted quantitation.

## Graphical abstract

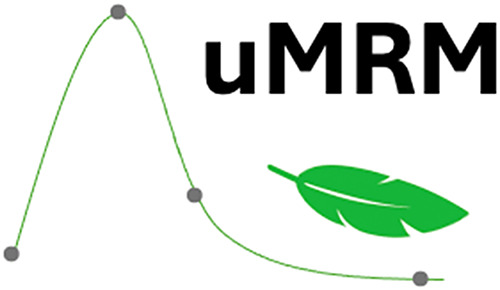

## Introduction

Reproducibility and quantitative robustness remain central challenges in metabolomics and lipidomics.^1–5^ Although high-resolution mass spectrometry (HRMS) enables broad molecular coverage, differences in instrumentation, acquisition strategies, and data-processing pipelines often limit interlaboratory consistency.^6–9^ Targeted multiple reaction monitoring (MRM) approaches provide highly reproducible and sensitive quantification,^10^ yet require predefined precursor–fragment transitions and prior analyte knowledge, restricting their utility in discovery-driven workflows.

Foundational work in metabolomics established analytical and computational strategies for global small-molecule profiling,^2–5^ and large-scale spectral resources have expanded molecular annotation capabilities.^8,9^ Several approaches have attempted to derive targeted MRM assays from untargeted MS/MS data;^11,20^ nevertheless, bridging untargeted discovery and quantitative deployment remains nontrivial. HRMS-based discovery generates large, vendor-specific data sets,^7–9^ whereas targeted MRM methods demand compound-specific optimization and authentic standards, increasing experimental burden.

To address this gap, we introduce untargeted/micro/universal multiple reaction monitoring (uMRM), a discovery-to-quantitation framework that converts high-resolution untargeted liquid chromatography–mass spectrometry/MS (LC–MS/MS) data into scheduled triple-quadrupole MRM transitions. Here, “micro” refers to minimal sample input and reduced data footprint during transition discovery, while “universal” reflects the empirical, vendor-agnostic generation of precursor–fragment transitions independent of proprietary optimization routines.

In this study, MS/MS spectra used for transition generation were acquired using stepped-energy data-dependent acquisition (DDA) following MS1 feature detection. Discrete collision-energy acquisitions (0, 10, 20, and 40 eV) provide empirical fragmentation data enabling systematic precursor–fragment transition generation. These data are processed through automated feature curation, including deisotoping, empirically validated in-source fragment (ISF) filtering, and spline-based collision-energy modeling to generate optimized MRM transitions.

Because transitions are scheduled using empirically observed retention times from pooled samples, nonlinear retention-time alignment is not required.^12–18^ The resulting vendor-agnostic transition tables enable quantitative deployment on triple-quadrupole instruments across representative biological matrices without reliance on authentic standards.

This framework provides a structured and reproducible pathway from untargeted discovery to targeted quantitation in metabolomics and lipidomics and may be extended to related molecular measurement domains.

## Methods

### Overall Experimental Design

All LC–MS and LC–MS/MS experiments were designed to integrate high-resolution discovery data with quantitative triple-quadrupole (QqQ) analysis. Pooled-sample data sets were first acquired using high-resolution LC–MS (MS^1^) to define the feature space, followed by data-dependent (DDA) LC–MS/MS acquisition to generate empirical fragmentation spectra for uMRM transition generation. These data were processed using a standalone uMRM analysis environment for feature curation, including deisotoping and in-source fragment (ISF) filtering,^19^ and spline-based collision-energy modeling. The resulting uMRM transitions were then implemented on QqQ instruments for quantitative analysis of representative biological matrices, including human urine and plasma-derived HDL/LDL lipid fractions. The uMRM workflow described here is platform-independent and does not inherently depend on proprietary spectral libraries for empirical transition generation for execution, although reference spectral libraries may be used for annotation and benchmarking where appropriate.

### Sample Preparation and Pooled Master Samples

For comprehensive feature coverage and to minimize bias, equal aliquots from all experimental groups were combined into pooled master samples. These composite samples served as the discovery data sets for DDA-based MS/MS acquisition at discrete collision energies, providing spectral reference points across the study.

Human plasma–derived HDL and LDL fractions (Kalen Biomedical) were diluted into lipoprotein-depleted serum (LPDS) media at 50 μg mL^–1^ and incubated with cultured cells for 36 h at 37 °C under 5% CO_2_. After incubation and centrifugation, cells were extracted with chilled isopropanol/water (4:1, v/v) and subjected to three freeze–thaw–sonication cycles to ensure complete recovery of polar and nonpolar lipids. Extracts were dried under vacuum and reconstituted in isopropanol/acetonitrile (1:1, v/v).

Urine was collected from healthy volunteers following oral administration of 200 mg ibuprofen. Samples were thawed on ice, combined into a pooled master sample, and extracted using 50:50 methanol/acetonitrile with 0.1% formic acid. Supernatants were evaporated to dryness and reconstituted in 50:50 water/methanol prior to LC–MS/MS analysis. Pooled and individual urine samples were analyzed in both ionization modes to capture ibuprofen and its major metabolites (hydroxy- and carboxy-ibuprofen).

To reduce matrix complexity and improve MS/MS sampling efficiency, pooled master samples were generated within defined experimental sets rather than across the entire study. Each experimental-set pool was analyzed independently for DDA-based transition discovery, and the union of resulting transitions was used for downstream quantitative MRM deployment. This strategy mitigates signal suppression in pooled analyses and increases the probability of sampling condition-specific features.

### LC–MS/MS Discovery Acquisition (QTOF)

Comprehensive high-resolution LC–MS (MS1) and DDA LC–MS/MS analyses were performed on Bruker Impact II and Agilent 6546 QTOF instruments coupled to UHPLC systems. Pooled samples were analyzed using reversed-phase LC over a mass range of *m*/*z* 100–1100 in both ionization modes.

High-resolution MS/MS spectra were acquired using DDA in a top-10 configuration (dynamic exclusion X s, intensity threshold Y, isolation width Z *m*/*z*) following MS1 feature detection. Discrete collision energies (0, 10, 20, and 40 eV) were applied to generate complementary fragmentation spectra for each precursor. Each collision energy was sampled as a distinct MS/MS event within a single LC–MS/MS run, consistent with the discrete-energy acquisition strategy validated in our prior MRM study.^21^

### Chromatographic Conditions

For lipidomic analyses (HDL/LDL data sets), mobile phases were:

- A: 50% H_2_O/30% ACN/20% IPA with 10 mM ammonium formate
- B: 90% IPA/9% ACN/1% H_2_O with 10 mM ammonium formate

Chromatography was performed on an ACQUITY BEH C18 column (1.0 mm × 100 mm, 1.7 μm) using a linear gradient from 10% to 100% B over approximately 15 min, followed by column re-equilibration. Source parameters: end-plate offset = 500 V, dry gas = 8 L min^–1^, dry temperature = 200 °C, nebulizer = 29 psi, and capillary voltage = 4000 V. Data were acquired at ∼10 Hz (MS) and 8 Hz (MS/MS).

For urine analyses (ibuprofen and metabolites), mobile phases were A: H_2_O with 0.1% formic acid and B: ACN. Chromatography was performed on an ACQUITY BEH C18 column (2.1 mm × 100 mm) using a gradient optimized for polar drug metabolites: 5% B at 0 min, 5% B at 4 min, 60% B at 10 min, 85% B at 18 min, followed by column wash and a 4 min re-equilibration. Source parameters in positive ion mode: end-plate offset = 500 V, dry gas = 11 L min^–1^, dry temperature = 200 °C, nebulizer = 20 psi, and capillary voltage = 4000 V. Data were acquired at ∼10 Hz (MS) and 8 Hz (MS/MS).

### MS/MS Data Annotation

Acquired DDA MS/MS spectra were compared against the METLIN tandem mass spectral database, which contains over 960,000 empirically acquired MS/MS spectra from authenticated chemical standards. METLIN reference spectra were collected using both direct infusion and LC–MS/MS methods in positive and negative ionization modes at four standardized collision energies (0, 10, 20, and 40 eV).

The METLIN database includes spectra acquired across multiple high-resolution mass spectrometry platforms, primarily Agilent quadrupole time-of-flight (QTOF) instruments, with additional contributions from Thermo Orbitrap, Bruker QTOF, and SCIEX QTOF systems. Only MS/MS spectra derived directly from authenticated reference standards were included; no in silico–predicted, extrapolated, or library-propagated spectra were used.

### Fragment Tracking and Collision-Energy Profiling

Fragment ions were dynamically tracked the top ten fragment ions across the four discrete collision energies (0, 10, 20, and 40 eV) using a ±0.5 Da grouping tolerance to associate recurring fragment ions derived from the same molecular feature across collision energies.^20,21^ This tolerance was used solely for cross-energy fragment alignment and not for precursor or fragment identity assignment. For each compound, MS/MS spectra were centroided and fragment alignment across collision energies was performed using an iterative *m*/*z* matching algorithm that prioritized consistent signal detection across at least three of the four collision energies. The spline fitting requires 3 energies however if fragments are not present across 3 or more it will still provide MRMs for the fragment at the CE observed

Each matched fragment group was assigned a collision energy–intensity profile, capturing the empirical change in fragment abundance as a function of collision energy. These CE-dependent profiles were used to construct continuous fragmentation trends through spline fitting (see Collision Energy Prediction and AI Refinement section). Fragments detected at only a single collision energy or exhibiting discontinuous or nonreproducible intensity behavior across energies were excluded from downstream modeling to improve robustness and reduce spurious fragment associations.

Quantifier and qualifier transitions were selected based on reproducibility, signal consistency, and collision-energy dependence across the CE range. The quantifier ion was defined as the most intense and stable fragment within the CE–intensity profile, while the qualifier ion was selected as the next most intense fragment meeting reproducibility criteria and a minimum mass separation of ≥2.0 Da from the precursor. This selection strategy ensured that transitions reflected empirically consistent, energy-dependent fragmentation behavior rather than single-point or instrument-specific noise.

### Collision Energy Prediction and AI Refinement

Collision energy (CE) predictions were generated using univariate spline fitting of fragment ion intensity as a function of collision energy (cubic splines; *k* = 2, smoothing factor *s* = 0). For each fragment, the predicted optimal CE corresponded to the maximum of the fitted spline curve. Predicted values below 5 eV or outside the experimentally sampled range were constrained to 5 eV, reflecting the lower operational limit of standard triple-quadrupole instruments.

Spline fitting constitutes the core CE prediction method in the uMRM framework. To assess robustness and generalizability across chemical classes, supervised regression models were applied to evaluate the relationship between empirically observed and spline-predicted CEs, as described in our prior Analytical Chemistry study.^21^ This AI BioSync framework was used to identify spline-fitting parameters that produced consistent CE predictions across structurally diverse compounds, rather than to replace empirical modeling.

Spline fitting and CE–intensity visualization were performed in R v4.3.2 using the *splines*, *mgcv*, and *ggplot2* packages. Regression analyses were implemented in Python v3.11 using *scikit-learn*. All analyses and final parameter selections were performed by the authors.

### Benchmarking and Validation

uMRM-derived transitions were benchmarked against experimentally optimized MRM transitions acquired on seven triple-quadrupole platforms, including Agilent 6495B, a second Agilent 6495, Agilent Ultivo, Waters Xevo TQ-XS, Waters Xevo TQS micro, Thermo TSQ Altis, and SCIEX API 3200 instruments. Performance was evaluated using three metrics: (1) fragment *m*/*z* agreement within ±0.5 Da, (2) collision energy prediction error (ΔCE) relative to empirically optimized values, and (3) overlap between uMRM-derived and manually optimized quantifier and qualifier transitions.

Benchmarking included 250 compounds spanning biologically and clinically relevant chemical classes, together with a mixture of more than 80 structurally diverse standards^21^ analyzed at concentrations of 1 μM and 1 nM to assess sensitivity. Across these data sets, uMRM-derived transitions showed strong agreement with experimentally optimized transitions and reproducible performance across instruments, supporting the accuracy and robustness of the transition-generation strategy for a total of over 300 compounds.

### Transition Generation and Curation

Feature tables and corresponding MS/MS spectra were processed using a dedicated uMRM analysis environment for isotope removal and in-source fragment (ISF) filtering.^19,22,23^ ISF filtering leveraged low-energy MS/MS data (including nominal 0 eV acquisitions) as empirical references for in-source fragmentation behavior, in combination with chromatographic coelution (±0.05 min), expected mass-difference relationships, and correlated intensity trends across collision energies.

In contrast to workflows that collapse all adduct species into a single neutral mass, uMRM treats adduct ions as analytically valid precursor species because adduct formation is often intrinsic to ionization chemistry and may represent the dominant, or sole, observable precursor for a given compound (e.g., certain carbohydrates and polar metabolites). Accordingly, adduct species were retained for transition generation when supported by consistent retention time, expected adduct mass relationships, and reproducible MS/MS behavior. When multiple precursor ions were determined to represent redundant adduct forms of the same underlying molecular feature, a single representative precursor ion was selected to avoid duplicate quantitation while preserving analytically meaningful ion species.

A practical distinction between uMRM and some HRMS feature workflows is that adduct ions are not treated as “noise” by default. Because many metabolites ionize preferentially as adduct species and may not yield robust [M + H]^+^ ions, preserving adduct precursors expands measurable chemical space while retaining empirical MS/MS validation.

Where reference data were available, curated precursor ions were cross-referenced against the METLIN tandem mass spectral library for annotation and benchmarking. This step was used solely for validation and did not form part of the core transition-generation workflow. Importantly, transition generation does not require METLIN matching; matched features were used only for annotation and benchmarking.

Spline fitting of fragment-ion intensity across the four discrete collision energies was performed within the AI BioSync framework to generate continuous collision-energy (CE) profiles. Quantifier and qualifier ions were automatically selected based on intensity maxima, *m*/*z* uniqueness (Δ ≥2 Da from the precursor), and reproducibility across at least three collision-energy steps. Fragment selection was based exclusively on empirically acquired stepped-energy MS/MS data and was not constrained to fragments present in matched reference spectra. Quantifier and qualifier selection is fully automated and rule-based; no manual curation was performed for transition selection.

When stepped-energy MS/MS data were acquired, precursor–fragment transitions were generated fully empirically from pooled-sample spectra without reliance on external spectral libraries. In cases where multi–collision energy MS/MS acquisition was not performed, previously curated METLIN MRM transitions could optionally be used to initialize quantifier and qualifier selection. Thus, empirical transition generation does not require METLIN for operation.

The impact of isotope removal and ISF filtering was evaluated by tracking feature counts at successive processing stages. For representative data sets, isotope consolidation produced modest reductions in feature counts, whereas subsequent ISF filtering reduced the remaining precursor features by approximately 60–75%, yielding curated precursor sets used for downstream transition generation.

### Quantitative Analysis on Triple Quadrupole Platforms

Empirically derived transitions were compiled into scheduled dynamic MRM methods and implemented on an Agilent 6495 triple-quadrupole mass spectrometer coupled to an Agilent 1290 UHPLC. Chromatographic separation was performed on a Waters BEH C18 column (1.0 mm × 100 mm, 1.7 μm) at a flow rate of 0.125 mL min^–1^ with 5 μL injection volumes. Positive and negative transitions were monitored within a single method using dynamic polarity switching.

Each compound was monitored using one quantifier and one or more qualifier transitions scheduled within ±0.3 min of the pooled-sample retention time. A constant 500 ms cycle time was maintained to ensure uniform temporal sampling across chromatographic peaks. Quantitative reproducibility was assessed using replicate injections and biological replicates.

In the ibuprofen study, hydroxy- and carboxy-ibuprofen metabolites were resolved across time points, while lipidomic analyses enabled quantitative monitoring of tocopherols, sterols, and phospholipids with high quantitative reproducibility across replicate analyses.

### Data Processing and Output Format

Acquired QqQ data were converted into standardized CSV files containing precursor and fragment *m*/*z*, polarity, optimized collision energy, retention time, and integrated peak area. These files, typically 100–200 kB in size—approximately 3 orders of magnitude smaller than corresponding high-resolution MS data sets—enable direct statistical analysis and efficient data sharing.

The resulting lightweight, vendor-agnostic format supports downstream analysis using either commercially available or freely available software platforms^17,18^ and facilitates long-term interoperability and reproducibility across instruments, laboratories, and studies.

### Software and Availability

All algorithms for fragment tracking, spline-based collision energy (CE) prediction, and transition selection were implemented in Python v3.11 using *pandas* (v2.2.2), *NumPy* (v1.26.4), *SciPy* (v1.13.1), and *scikit-learn* (v1.4.0), with additional analyses performed in R v4.3.2 using the *splines*, *mgcv*, and *ggplot2* packages. Jupyter Notebooks were used for pipeline development, visualization, and quality control.

The uMRM workflow described here is platform-independent. Representative pooled-sample mzML files (full-scan MS and stepped-energy MS/MS), curated precursor tables, and uMRM-derived transition lists (CSV format) are provided in the Supporting Information. Reference spectral libraries may be used for annotation and benchmarking where appropriate but are not required for execution of the uMRM workflow.

Example scripts demonstrating spline-based collision-energy modeling and automated quantifier/qualifier transition selection are provided in in the Supporting Information.

## Results and Discussion

### Overview of the uMRM Framework

The uMRM framework bridges high-resolution untargeted discovery and quantitative targeted analysis by converting experimentally acquired high-resolution LC–MS and DDA LC–MS/MS data into precursor–fragment transitions suitable for triple-quadrupole MRM workflows. uMRM converts high-resolution discovery data into scheduled MRM transitions suitable for triple-quadrupole quantitation (Figure 1).

**Figure 1:**
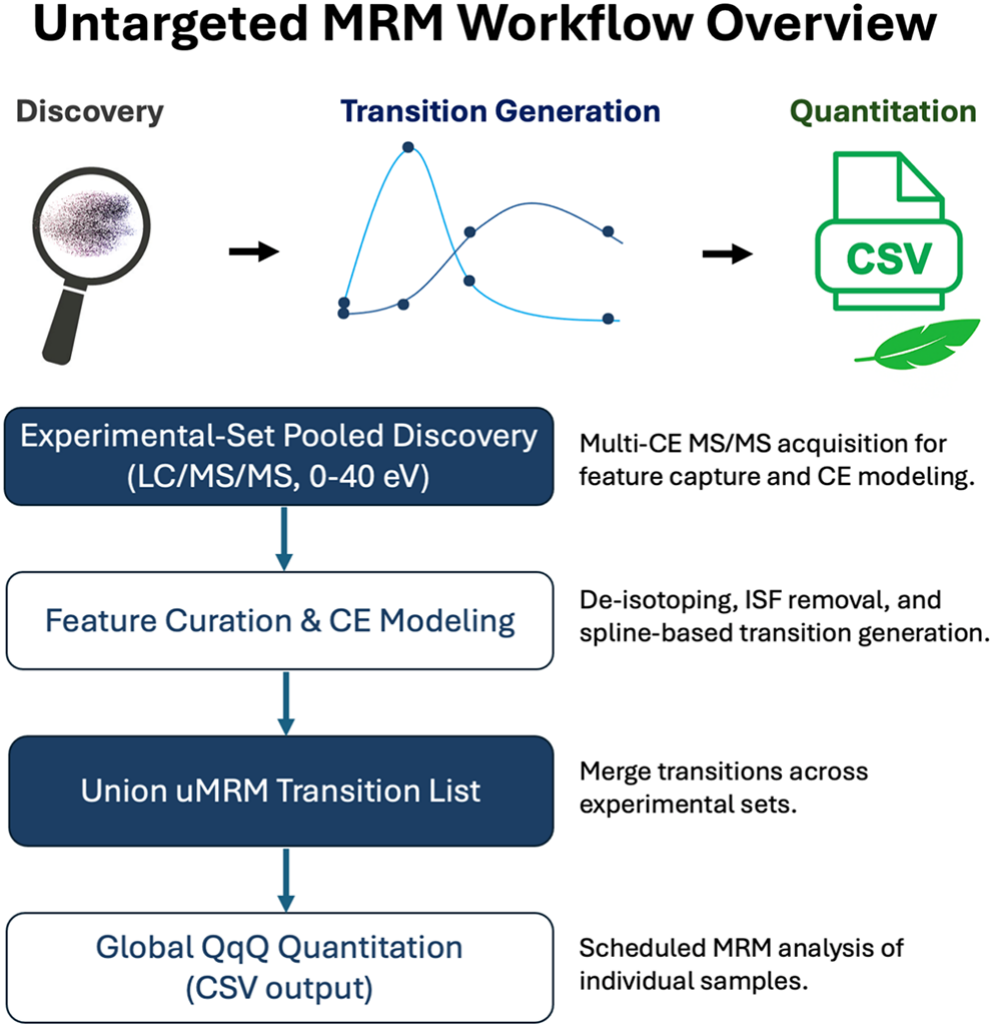
Overview of the uMRM workflow linking high-resolution discovery to quantitative MRM analysis. Experimental-set pooled samples are analyzed by LC–MS and multi–collision energy LC–MS/MS (0, 10, 20, and 40 eV) to capture representative precursor and fragment information. Detected features undergo automated curation, including deisotoping and empirically validated in-source fragment (ISF) filtering, followed by collision-energy modeling to generate optimized precursor–fragment transitions. Transition generation operates in both reference-guided and fully empirical modes. Transitions derived across experimental sets are merged into a unified list and deployed as a scheduled (dynamic) MRM method on triple-quadrupole instruments for quantitative analysis of individual samples. Quantitative outputs are exported as vendor-agnostic CSV tables for relative comparative analysis.

The workflow begins with analysis of a pooled master sample by high-resolution LC–MS and LC–MS/MS. To ensure reproducibility and consistency with prior validation, MS/MS data are acquired at four discrete collision energies (0, 10, 20, and 40 eV), generating complementary fragmentation spectra that capture low- and moderate-energy dissociation behavior across chemically diverse analytes. These pooled-sample acquisitions provide a comprehensive empirical map of precursor and fragment ions representative of the full study.

Extracted features are subjected to automated deisotoping and in-source fragment (ISF) filtering, yielding a curated set of empirically supported precursor features. Each curated precursor is then associated with its corresponding MS/MS spectra across collision energies, forming precursor-centric data sets suitable for downstream transition modeling. The quantitative impact of these curation steps is evaluated explicitly using experimental data sets, as described below.

Retaining analytically supported adduct precursor ions expanded the number of unique precursor–fragment transitions available for downstream MRM scheduling. In both HDL/LDL and urine data sets, a substantial fraction of curated precursor features corresponded to nonprotonated adduct species, underscoring the importance of preserving adduct chemistry rather than collapsing all features to a single neutral mass.

Fragment-ion intensities across collision energies are modeled using spline-based approaches, enabling empirical characterization of energy–response relationships for each precursor–fragment pair. From these models, quantifier and qualifier ions are selected based on signal stability, fragmentation consistency, and collision-energy dependence. This empirically grounded strategy eliminates the need for authentic standards or purely predictive fragmentation models while preserving quantitative robustness.

The resulting transition sets are deployed on triple-quadrupole instruments as scheduled (dynamic) MRM methods, with transitions anchored to retention times observed directly in pooled samples. This empirical retention-time anchoring avoids nonlinear alignment and postacquisition correction, simplifying quantitative analysis and improving reproducibility across runs. Through this approach, uMRM combines the sensitivity and robustness of MRM with the breadth of high-resolution untargeted discovery in a platform-agnostic and scalable framework.

While the uMRM workflow is currently implemented within the dedicated analysis environment scripps.umrm.edu, the underlying methodological steps, feature curation, fragment filtering, spline-based collision-energy optimization, and transition export, are described in sufficient detail to enable conceptual replication in alternative computational environments.

### An Empirical Strategy for the Removal of In-Source Fragments

Feature curation is a critical step for ensuring transition specificity and quantitative reproducibility within the uMRM framework. Pooled-sample LC–MS (MS^1^) data and DDA LC–MS/MS spectra acquired at four discrete collision energies (0, 10, 20, and 40 eV) were first processed to consolidate isotopic variants and redundant ion forms into unique, precursor-centric features prior to transition modeling.

In-source fragment (ISF) removal was then performed to distinguish intact molecular precursors from fragment ions generated upstream of the collision cell. This process was based on an empirically validated strategy consistent with our prior work demonstrating that in-source fragmentation closely mirrors low-energy MS/MS behavior.^19^ Importantly, MS/MS spectra acquired at nominal 0 eV were **not** assumed to be fragment-free; instead, they were used as a low-energy reference to identify fragment ions that persist even in the absence of intentional collision-induced dissociation.

ISF filtering leveraged the convergence of three empirical criteria to identify **ISF-derived precursor features**:

1. **Accurate-mass association:** fragment-associated ion features were linked to precursor features based on high mass accuracy (±5–10 ppm mass difference criteria), without assuming specific fragmentation pathways.
2. **Chromatographic coelution:** fragment-associated ion features coeluted with their corresponding precursor features within a narrow retention-time window (±0.05 min), consistent with a shared chemical origin.
3. **Collision-energy dependence:** fragment-associated ion features were detected at 0 eV and persisted or increased in intensity at higher collision energies (10–40 eV), behavior characteristic of fragment-derived rather than intact molecular ions.

Features meeting these criteria were classified as **ISF-derived precursor features** and excluded from subsequent transition modeling. This filtering step prevents fragment-derived MS^1^ features from propagating into downstream MRM transition lists, where they would otherwise lead to nonspecific or misleading quantitative targets.

The quantitative impact of each curation step is summarized in Table 1 using representative HDL/LDL and urine data sets.

**Table 1:** Quantitative Impact of Feature Curation Steps in the uMRM Workflow^a^

data set		features with MS/MS	after isotope consolidation	features after ISF removal
HDL/LDL		5405	4630	1613
urine		3145	3116	795

a Feature counts are shown for representative HDL/LDL and urine datasets at successive stages of curation. “Features with MS/MS” denote precursor features for which tandem MS spectra were acquired. The modest reduction following isotope consolidation reflects the limited selection of isotopic peaks for MS/MS acquisition, whereas in-source fragment (ISF) removal substantially reduces the number of fragment-derived precursor features propagated into downstream MRM transition generation.

Following feature curation and ISF removal, 29% (*n* = 467) of curated precursor features in the HDL/LDL data set were matched to METLIN reference spectra using the criteria described above. In contrast, a substantially smaller fraction of urine features were annotated (8%), consistent with the chemical complexity of urine and the prevalence of structurally uncharacterized endogenous and xenobiotic metabolites. Unmatched features were retained as empirically derived precursors for uMRM transition generation.

The limited numerical impact of deisotoping reflects a fundamental property of DDA-based MS/MS acquisition, in which isotopic peaks are not selected at appreciable numbers for fragmentation and therefore contribute minimally to MS/MS-level redundancy. Following ISF removal, the remaining feature set consisted of authentic molecular precursors that fragmented only under controlled collision-energy conditions. These curated precursors were carried forward for spline-based modeling of fragment-ion energy–response relationships and for selection of quantifier and qualifier transitions. As illustrated in Figure 2, empirical removal of coeluting ISF artifacts substantially improved precursor–fragment specificity, reduced propagation of fragment-derived targets, and enhanced signal reproducibility across replicate analyses.

**Figure 2:**
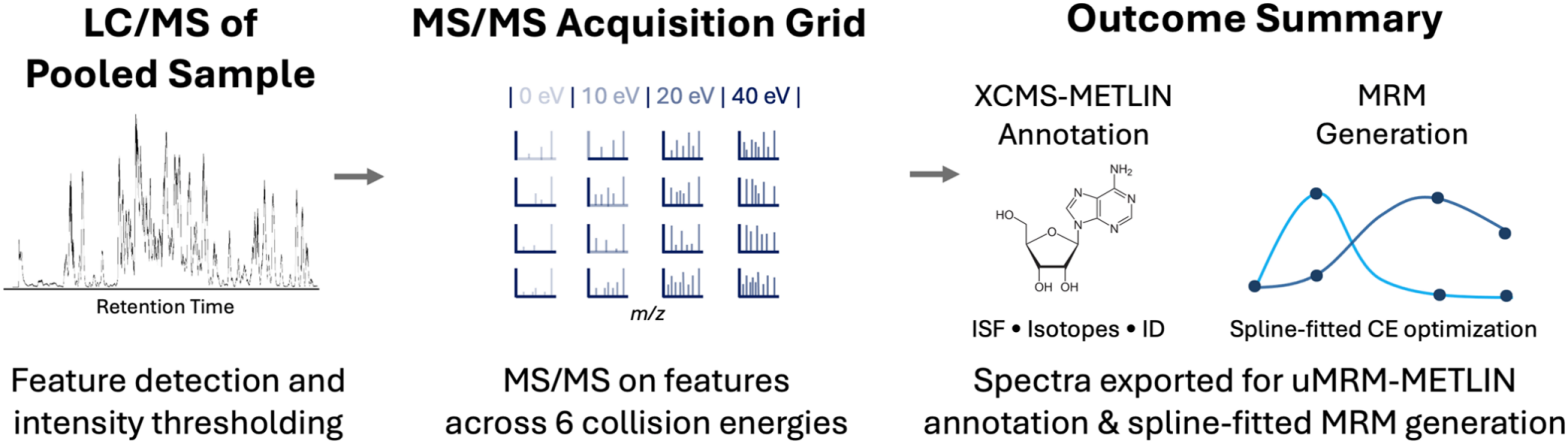
Pooled-sample LC/MS and LC-MS/MS acquisition and empirical transition generation for uMRM. A pooled master sample is analyzed by LC–MS to detect and threshold molecular features, followed by discrete-energy LC–MS/MS acquisition (0, 10, 20, and 40 eV) to capture complementary fragmentation behavior for each precursor. The resulting multienergy MS/MS spectra are subjected to automated feature curation, including deisotoping, and empirically validated in-source fragment (ISF) filtering. Curated precursor–fragment relationships are then used to construct spline-fitted collision-energy (CE) profiles, enabling empirical selection and optimization of quantifier and qualifier MRM transitions. Transition generation can proceed in a reference-guided mode when high-confidence library matches are available, or in a fully empirical mode using pooled-sample data alone. The resulting transitions form the basis for downstream scheduled MRM analysis.

This curation strategy ensures that uMRM transitions represent chemically meaningful and reproducible molecular events, rather than source- or matrix-dependent fragmentation artifacts.

### Spline-Based Transition Optimization

Following feature curation, precursor–fragment transitions were optimized using the spline-based collision-energy (CE) modeling framework described in the Methods. Briefly, fragment-ion intensities acquired at four discrete collision energies (0, 10, 20, and 40 eV) were used to construct empirical CE–response profiles for each precursor–fragment pair (Figure 3). The resulting models enabled selection of quantifier and qualifier transitions based on signal stability, fragmentation consistency, and CE-dependent behavior.

**Figure 3:**
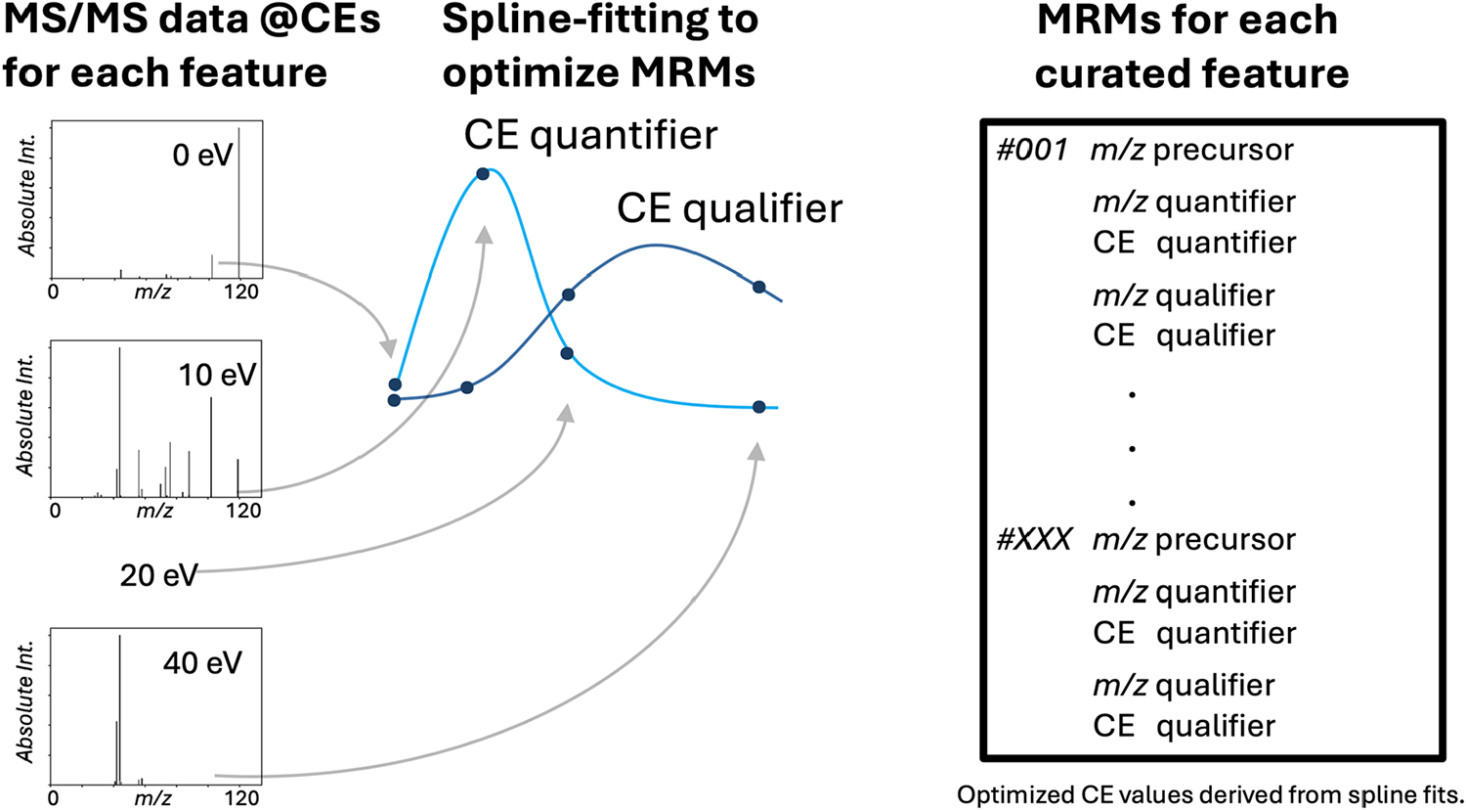
Spline-based optimization of MRM transitions from discrete-energy MS/MS data. High-resolution MS/MS spectra acquired at discrete collision energies (0, 10, 20, and 40 eV) are used to characterize fragment-ion intensity as a function of collision energy for each curated precursor. Cubic spline fitting is applied to these empirical measurements to generate continuous collision-energy (CE) response curves that capture fragmentation efficiency and stability. For each precursor–fragment pair, the spline-fitted intensity maximum is used to define the optimal collision energy for quantifier ions, while additional fragments exhibiting orthogonal *m*/*z* values and consistent energy dependence are selected as qualifiers. The resulting optimized transitions—defined by precursor *m*/*z*, fragment *m*/*z*, and collision energy—are compiled into a transition list for downstream scheduled MRM analysis.

For each precursor, the optimal collision energy was defined by the modeled intensity maximum of the selected quantifier ion, while additional fragments exhibiting orthogonal *m*/*z* values and reproducible energy dependence were designated as qualifiers.^20,21^ Fragment ions displaying irregular or nonreproducible CE profiles were excluded from transition selection, improving robustness and minimizing propagation of unstable fragmentation events.

The final uMRM transition set for each precursor included precursor *m*/*z*, fragment *m*/*z*, optimized collision energy, and empirically observed retention time, forming the basis for scheduled triple-quadrupole deployment.

To evaluate cross-platform robustness, uMRM-derived collision energies were compared with independently optimized MRM methods across seven triple-quadrupole platforms (*n* = 307 compound analyses). The overall median ΔCE (CE_uMRM – CE_optimized) was −1 eV, indicating no systematic directional bias, while the median absolute deviation was 5 eV (Table 2). Across all platforms, 74% of transitions were within ±10 eV of optimized values, demonstrating strong concordance between empirically modeled and manually optimized collision energies.

**Table 2:** Empirical Validation of uMRM Collision-Energy Optimization^a^

metric	Agilent	Thermo	Waters	Sciex	overall
N	217	63	21	6	307
median ΔCE (eV)	0	–3	–5	–6	–1
median |ΔCE| (eV)	5	6	9	11	5
within ±5 eV	55%	44%	33%	33%	51%
within ±10 eV	74%	76%	67%	50%	74%

a ΔCE was defined as CE_uMRM – CE_optimized for the quantifier ion. Median ΔCE reflects directional bias, whereas median |ΔCE| represents the typical magnitude of deviation irrespective of sign. Percentages indicate the fraction of transitions within ±5 eV and ±10 eV of optimized values. Results are stratified by instrument platform and summarized overall.

These results indicate that spline-modeled CE selection provides accurate and transferable transition optimization across chemically diverse analytes and instrument platforms.

### Spline Fitting Scenarios: Reference-Guided versus Empirical Discovery Modes

The uMRM framework supports two complementary operational modes, both relying on the same empirical CE-modeling strategy described above.

When a precursor ion could be confidently matched to a reference spectrum, a reference-guided mode was applied. In this case, previously validated fragment assignments served as an initial guide, while empirical CE modeling refined transition selection under the specific experimental conditions of the study. This approach preserves continuity with established reference data while enabling matrix- and instrument-specific optimization.

For precursor ions lacking reference annotations, an empirical discovery mode was used. Here, transition selection relied solely on pooled-sample MS/MS data, without structural annotation or prior spectral knowledge. Quantifier and qualifier ions were selected based on reproducible CE-dependent behavior and signal stability, enabling transition generation for structurally uncharacterized features.

Importantly, both modes operate within the same empirical modeling framework and require no in silico fragmentation prediction or authentic standards. Reference databases may support annotation and benchmarking but are not required for transition generation. This unified strategy enables consistent transition optimization across annotated and unannotated molecular entities within a single data-driven workflow.

### Quantitative Application and Dynamic MRM Performance

The empirically optimized uMRM transition set was implemented as a scheduled (dynamic) MRM method for quantitative analysis across biological and chemical matrices (Figure 4). Each analyte was monitored using one quantifier and one or more qualifier transitions within narrow retention-time (RT) windows (typically ± 0.3 min), maximizing dwell time and signal-to-noise while maintaining constant cycle times (∼500 ms). Scheduling based on empirically observed pooled-sample retention times eliminated the need for nonlinear postacquisition alignment and reduced susceptibility to chromatographic drift.

**Figure 4:**
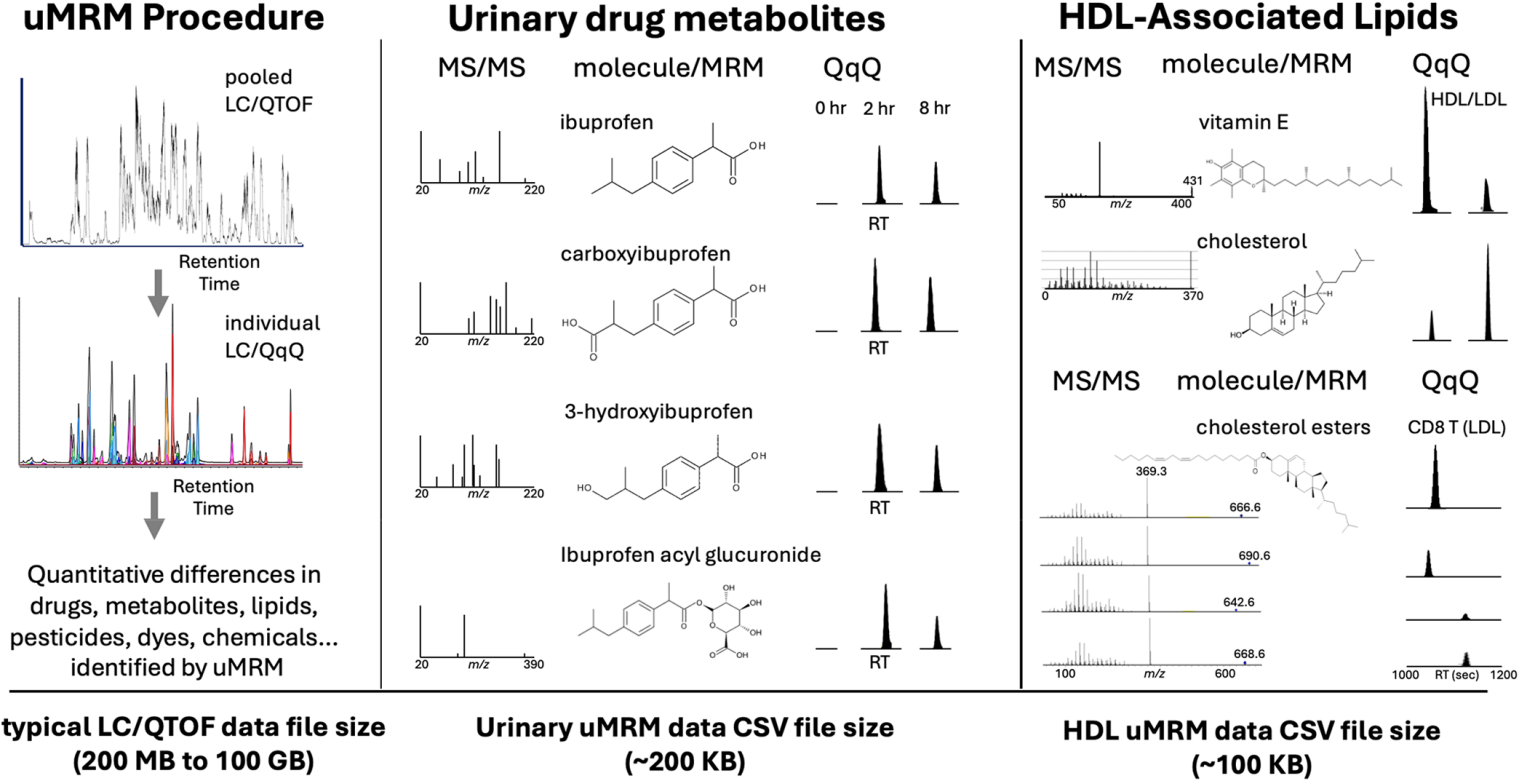
Application of uMRM to drug metabolism and lipidomics with dynamic MRM quantitation. The uMRM workflow integrates pooled-sample LC/QTOF discovery data with targeted LC/QqQ analysis to enable label-free quantitative measurements across diverse chemical classes. **Left:** pooled LC/QTOF data are used to derive spline-optimized MRM transitions, which are subsequently applied to individual LC/QqQ analyses to quantify drugs, metabolites, lipids, and other small molecules. **Center:** uMRM-based detection and relative quantification of ibuprofen and its metabolites (carboxy-, hydroxy-, and acyl glucuronide derivatives) in human urine, demonstrating temporal resolution following oral administration. **Right:** uMRM quantification of HDL-associated lipids, including vitamin E, cholesterol, and cholesterol esters, from lipoprotein and immune cell extracts, illustrating applicability to translational lipidomics. Across applications, uMRM compresses discovery-scale LC/QTOF data sets (typically 200 MB–100 GB per file) into lightweight, vendor-agnostic CSV outputs (∼100–200 kB) suitable for direct statistical and comparative analysis.

Quantitative reproducibility was evaluated using replicate injections and representative biological samples. In the ibuprofen metabolism study, uMRM resolved multiple hydroxy- and carboxy-ibuprofen metabolites and captured consistent temporal trends following oral administration without reliance on authentic standards. In a lipidomics application, pooled HDL and LDL fractions were analyzed using the same spline-optimized transition set, enabling reproducible quantification of tocopherols, sterols, and phospholipids across replicate analyses. Vitamin E, cholesterol, and cholesterol esters represented high-confidence METLIN matches (Level 1/2), supported by accurate mass (<5 ppm), characteristic MS/MS fragmentation, and expected chromatographic behavior. These examples demonstrate that transitions derived from a single pooled discovery data set can support quantitative analysis across chemically diverse analytes.

Quantitative outputs were exported as vendor-agnostic CSV files containing precursor and fragment *m*/*z*, optimized collision energy, retention time, and integrated peak areas. These files (typically 100–200 kB) are orders of magnitude smaller than corresponding high-resolution LC–MS data sets while preserving explicit precursor–fragment traceability, enabling efficient downstream statistical analysis and data sharing.

In cases where distinct molecular features share identical precursor–fragment pairs, specificity is maintained through pooled-sample retention-time anchoring, narrow RT scheduling windows, qualifier-ion confirmation, and transition ratio consistency. Although structural isomers with identical fragmentation patterns may remain indistinguishable, this limitation reflects inherent constraints of MRM-based quantification rather than a property unique to uMRM.

Because DDA acquisition prioritizes higher-intensity precursors under top-N selection, not all curated MS1 features are fragmented during discovery. In the present data set, 10% of curated MS1 features were triggered for MS/MS under the applied acquisition settings. Features not selected for MS/MS were therefore not eligible for transition generation in the current workflow. Accordingly, uMRM transition generation is limited to empirically fragmented precursors, representing a practical constraint of DDA-based discovery rather than a limitation specific to the uMRM framework. uMRM does not claim that DDA improves fragmentation efficiency for low-abundance precursors during discovery; rather, the sensitivity advantages of the framework arise during the quantitative triple-quadrupole deployment phase once precursor–fragment pairs have been empirically defined.

Sensitivity advantages arise during the quantitative deployment phase. Once precursor–fragment pairs are defined, triple-quadrupole MRM detection is widely recognized to provide improved signal-to-noise and dynamic range in targeted applications relative to full-scan HRMS1 acquisition. For very low-intensity precursors, fragment-ion prioritization based on reproducible and high-intensity collision-energy profiles enhances quantitative robustness. In some cases—particularly for large or highly apolar lipids—only a single reproducible fragment may be observed, resulting in a single quantifier transition, consistent with conventional targeted lipidomics practice.

Overall, uMRM provides a transparent and grounded pathway from untargeted discovery to sensitive, selective quantitative measurement across chemically diverse analytes.

### uMRM.scripps.edu (β) Platform and Interoperability

To support reproducibility and provide a reference implementation of the uMRM framework, a β version of a dedicated analysis environment (uMRM.scripps.edu) has been developed (Figure 5). This platform implements the workflow described in this manuscript, enabling conversion of pooled-sample high-resolution LC–MS/MS data into empirically derived precursor–fragment transition tables.

**Figure 5:**
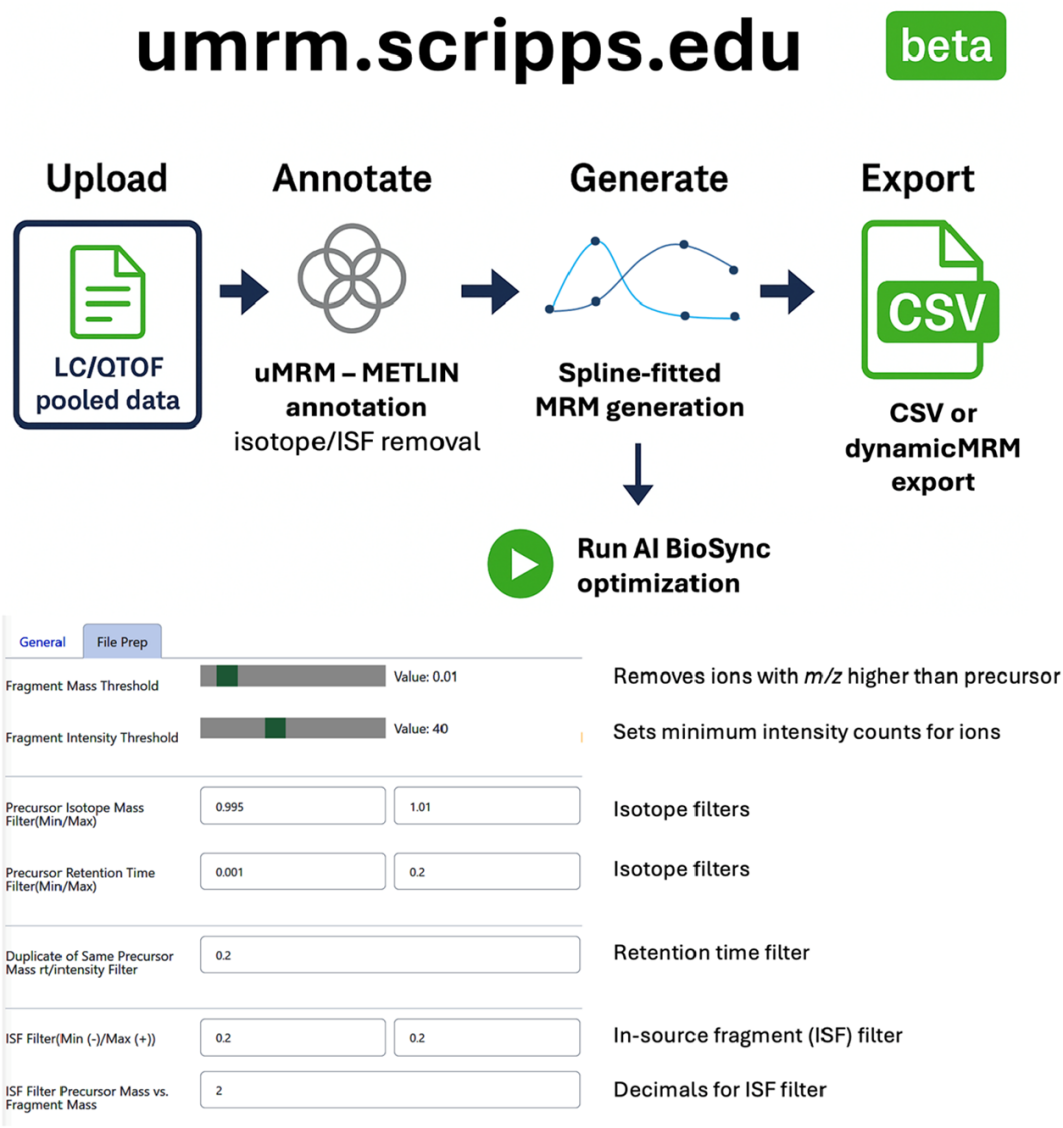
β implementation of the uMRM workflow for transition generation and export. A β implementation of the uMRM workflow (uMRM.scripps.edu) was developed to support automated generation of untargeted MRM transitions from pooled-sample LC/QTOF data. The platform enables upload of discovery-phase MS and MS/MS data, automated feature curation including isotope and in-source fragment (ISF) filtering, and spline-based modeling of collision-energy–dependent fragmentation to derive optimized MRM transitions. The resulting transition lists can be exported as vendor-agnostic CSV files or formatted for dynamic MRM acquisition on triple-quadrupole instruments. Parameter controls allow users to define filtering thresholds and curation criteria consistent with the analytical procedures described in this study. The β implementation is provided as a reference environment to illustrate workflow execution and does not represent a required dependency for applying the uMRM methodology.

The reference implementation automates key steps including isotope consolidation, in-source fragment (ISF) filtering, spline-based modeling of fragment-ion intensity across discrete collision energies, and selection of optimized quantifier and qualifier transitions. All algorithmic procedures correspond directly to those described in the Methods.

The resulting outputs are vendor-agnostic CSV transition tables containing precursor *m*/*z*, fragment *m*/*z*, optimized collision energy, retention time, and polarity. These lightweight files preserve explicit precursor–fragment relationships and are directly deployable on triple-quadrupole platforms or suitable for downstream statistical analysis and data interpretation.^24–26^

The web environment serves as a transparent demonstration of the uMRM workflow and facilitates reproducible application of the methodology. Representative data sets and transition tables are publicly deposited to enable independent evaluation of feature curation and transition generation.

## Conclusion

Untargeted/micro/universal multiple reaction monitoring (uMRM) provides an empirically driven workflow that links high-resolution untargeted discovery with quantitative triple-quadrupole analysis. By deriving precursor–fragment transitions directly from pooled-sample stepped-energy MS/MS data and modeling collision-energy–dependent fragmentation behavior, uMRM enables automated generation of optimized MRM transitions without reliance on in silico prediction or extensive compound-specific optimization.

Through isotope consolidation, empirically validated in-source fragment filtering, and retention-time anchoring, uMRM converts untargeted LC–MS/MS features into scheduled, vendor-agnostic transition tables suitable for quantitative deployment. Once empirically defined, these transitions can be implemented on triple-quadrupole platforms to provide sensitive and selective measurement across diverse chemical classes.

Although transition discovery inherits the practical constraints of DDA-based MS/MS acquisition, the framework provides a structured and reproducible approach for converting discovery-scale data sets into targeted quantitative methods. This strategy may be applicable to related domains where empirical fragmentation data can inform quantitative transition development.

## Supplementary Material





## Notes

Representative benchmarking data and example code supporting the reproducible, rule-based transition generation framework underlying uMRM (PDF)
uMRM vs Trad MRM (CSV)
