# Supplementary material for: Untargeted Multiple Reaction Monitoring
Source: Anal Chem. 2026 Mar 17;98(12):8985–94. doi: 10.1021/acs.analchem.5c06838 (PMC13044877; doi:10.1021/acs.analchem.5c06838)
Supplement: Supplementary file 1 [file ac5c06838_si_001.pdf]

Supplemental Material

Untargeted Multiple Reaction Monitoring (uMRM)

Winnie Uritboonthai[1], Aries Aisporna[1], Linh Hoang[1], Bill Webb[1], Elizabeth M. Billings[1], Corey Hoang[1], Mirna Tobea[1], Chelsea C. Cates-Gatto[2], Amanda J. Roberts[3], Tony Teav[4], Rebecca Borreggine[4], Ioana Kyritsi[4], Hector Gallart-Ayala[4], Julijana Ivanisevic[4], Anna Popova[5], James R. Williamson[5,6], Robert Plumb[7*], and Gary Siuzdak[1,5*]

[1]*Scripps Center for Metabolomics and Mass Spectrometry, The Scripps Research Institute, La Jolla, California 92037, United States*

[2]*Gnotobiotic and Cryobank Facility, The Scripps Research Institute, La Jolla, CA, 92037, USA*

[3]*Animal Models Core Facility, The Scripps Research Institute, La Jolla, CA, 92037, USA*

[4]*Metabolomics and Lipidomics Platform, Faculty of Biology and Medicine, University of Lausanne, Lausanne, Switzerland*

[5]*Department of Integrative Structural and Computational Biology, Department of Chemistry, The Scripps Research Institute, La Jolla, CA, 92037, USA*

[6]*The Skaggs Institute for Chemical Biology, The Scripps Research Institute, La Jolla, CA 92037*

[7]*Centre for Metabolomics Research, University of Liverpool, Liverpool, UK, L69 7ZB*

*Authors to whom correspondence should be addressed:
robert.plumb@liverpool.ac.uk and siuzdak@scripps.edu

Overview
This Supporting Information provides representative benchmarking data and example code supporting the reproducible, rule-based transition generation framework underlying uMRM.
The materials included here:

- Illustrate spline-based collision-energy (CE) modeling
- Demonstrate automated quantifier and qualifier selection
- Provide cross-platform benchmarking examples
- Enable independent reproduction of transition-generation logic

All materials operate on vendor-neutral input tables and do not require access to the web-based uMRM implementation.

Reviewer-Access Data Archive
Representative pooled LC–MS and stepped-energy LC–MS/MS datasets used for transition discovery are provided for reviewer access via a temporary archive:
Reviewer-access archive:
https://www.dropbox.com/scl/fi/fmsl5xjlum3bv9xa6qkw3/uMRM-Data-For-Review.zip?rlkey=5f72qw8qhbmtpnb7nbupupv0j&dl=0
The archive includes:

- Representative pooled-sample mzML files
- Example precursor/fragment intensity tables used for spline modeling
- Supporting files required to reproduce the transition-generation workflow

These materials are provided for transparency during peer review. Upon publication, representative datasets will be deposited in a permanent community repository (e.g., Metabolomics Workbench or MetaboLights) and assigned a public accession number. This reviewer-access archive is provided for transparency during peer review and is not intended as permanent public hosting.

Included Files

1. Transition Benchmarking Dataset
File:
2026-02-25) uMRM vs Trad MRM.csv
This dataset contains 307 precursor–fragment transitions derived using the uMRM workflow and benchmarked against experimentally optimized QqQ MRM transitions acquired across seven instruments representing four manufacturers.
The table includes:

- Compound identifier
- Instrument platform
- Precursor m/z
- Fragment m/z
- Optimized collision energy

This file demonstrates cross-platform transferability and agreement between empirically derived uMRM transitions and traditionally optimized MRM transitions.

---

2. Supplementary Code S1 — Example Transition Generation Script (Python)
This Supplementary Code provides an illustrative, minimal implementation of spline-based collision-energy modeling and automated quantifier/qualifier transition selection.
The script demonstrates:

- Fragment tracking across discrete collision energies
- Spline-based modeling of fragment intensity vs collision energy
- Automated quantifier and qualifier selection
- Collision-energy optimization

This code represents the core rule-based logic described in the Methods section. It is provided for methodological transparency and reproducibility.

Algorithmic Summary
Transition generation follows the rule-based steps described in the manuscript:
1. Fragment Tracking Across Collision Energies
Fragment ions are matched across discrete CE acquisitions. Intensities are tracked per CE.
2. Precursor Proximity Filtering
Fragments within ±2 Da of the precursor m/z are excluded to remove precursor-related and in-source fragment artifacts.
3. Spline-Based CE Optimization
A quadratic spline (UnivariateSpline, k = 2) is fitted to fragment intensity vs CE.

- Optimal CE is predicted from the spline maximum.
- If insufficient CE points exist, the CE with maximal observed intensity is selected.

4. Automated Transition Selection

- Quantifier ion: highest-intensity non-redundant fragment
- Qualifier ions: next most intense fragments (when available)
- No manual curation is performed

These procedures correspond directly to the rule-based methodology described in the revised manuscript.

Supplementary Code S1
Scope and Purpose
This code provides a minimal Python implementation of uMRM transition generation.
It demonstrates:

1. Spline-based modeling of fragment intensity as a function of collision energy
2. Automated, rule-based selection of quantifier and qualifier transitions
3. Export of vendor-neutral transition tables suitable for QqQ method development

It does not represent the full raw-data preprocessing pipeline, web implementation, or database-matching infrastructure.

Expected Input Format

The input CSV should contain the following columns:

- precursor_mz
- fragment_mz
- collision_energy
- intensity

Each row represents one fragment intensity measured at one collision energy.

Dependencies

Python ≥ 3.9

Required packages:

- pandas
- numpy
- scipy

Installation example:

pip install pandas numpy scipy

Supplementary Code S1 (Python)

```
import argparse
import numpy as np
import pandas as pd
from scipy.interpolate import UnivariateSpline


def spline_fit(ces, intensities):
    """
    Fit quadratic spline (k=2) to intensity vs collision energy.
    Returns CE corresponding to maximum predicted intensity.
    Falls back to observed maximum if insufficient data.
    """
    ces = np.array(ces, dtype=float)
    intensities = np.array(intensities, dtype=float)

    mask = ~np.isnan(ces) & ~np.isnan(intensities)
    ces = ces[mask]
    intensities = intensities[mask]

    if len(ces) < 3:
        return float(ces[np.argmax(intensities)])

    try:
        spl = UnivariateSpline(ces, intensities, k=2, s=0)
        grid = np.linspace(np.min(ces), np.max(ces), 200)
        pred = spl(grid)
        return float(grid[np.argmax(pred)])
    except Exception:
        return float(ces[np.argmax(intensities)])


def select_transitions(
    df,
    precursor_mz_col="precursor_mz",
    fragment_mz_col="fragment_mz",
    ce_col="collision_energy",
    intensity_col="intensity",
    precursor_exclusion_da=2.0,
):
```

```python
    """
    Automated rule-based transition selection:
    - Exclude fragments within ± precursor_exclusion_da of precursor m/z
    - Fit spline for each precursor-fragment pair
    - Select quantifier and qualifier transitions automatically
    """

    transitions = []
    grouped = df.groupby([precursor_mz_col, fragment_mz_col], dropna=False)

    tmp = []

    for (prec_mz, frag_mz), g in grouped:
        if pd.isna(prec_mz) or pd.isna(frag_mz):
            continue

        if abs(float(frag_mz) - float(prec_mz)) < precursor_exclusion_da:
            continue

        ces = g[ce_col].values
        ints = g[intensity_col].values

        opt_ce = spline_fit(ces, ints)
        max_int = float(np.nanmax(ints))

        tmp.append(
            {
                "precursor_mz": float(prec_mz),
                "fragment_mz": float(frag_mz),
                "opt_ce": float(opt_ce),
                "max_intensity": max_int,
            }
        )

    if len(tmp) == 0:
        return pd.DataFrame(
            columns=["precursor_mz", "fragment_mz", "opt_ce", "role"]
        )

    tmp_df = pd.DataFrame(tmp)

    for prec_mz, g in tmp_df.groupby("precursor_mz"):
        g = g.sort_values("max_intensity", ascending=False).reset_index(drop=True)

        g.loc[0, "role"] = "quantifier"

        if len(g) > 1:
            g.loc[1:, "role"] = "qualifier"

        transitions.append(
            g[["precursor_mz", "fragment_mz", "opt_ce", "role"]]
        )

    return pd.concat(transitions, ignore_index=True)


def main():
    parser = argparse.ArgumentParser(
        description="uMRM transition generation example script"
    )
    parser.add_argument("--infile", required=True)
    parser.add_argument("--outfile", required=True)
    args = parser.parse_args()

    df = pd.read_csv(args.infile)
    out = select_transitions(df)
    out.to_csv(args.outfile, index=False)
```

```
if __name__ == "__main__":
    main()
```

Intended Scope

This Supporting Information demonstrates:

- Reproducible transition-selection logic
- Vendor-neutral implementation
- Independence from proprietary infrastructure

It does not include:

- Full raw-data preprocessing pipelines
- Web-platform implementation code
- METLIN matching/scoring infrastructure
- Database connectivity components

The purpose is to provide transparent documentation of the core uMRM transition-generation methodology described in this study.
